# Causal relationship between ischemic stroke and its subtypes and frozen shoulder: a two-sample Mendelian randomization analysis

**DOI:** 10.3389/fneur.2023.1178051

**Published:** 2023-05-18

**Authors:** Xiaofeng Lv, Zeng Hu, Fangqi Liang, Shanshan Liu, Haiping Gong, Jihang Du, Xinmin Deng, Jun-Hui Qian, Qian Nie, Jian Luo

**Affiliations:** ^1^Hospital of Chengdu University of Traditional Chinese Medicine, Chengdu, Sichuan, China; ^2^School of Acupuncture and Tuina, Chengdu University of Traditional Chinese Medicine, Chengdu, China; ^3^Shenzhen Hospital of Beijing University of Chinese Medicine (Longgang), Shenzhen, China; ^4^Guang'an Traditional Chinese Medicine Hospital, Guang'an, Sichuan, China

**Keywords:** Mendelian randomization, ischemic stroke, frozen shoulder, genome-wide association study, causal association

## Abstract

**Background:**

Previous epidemiological and other studies have shown an association between ischemic stroke (IS) and frozen shoulder (FS). However, the causal relationship between them remains unclear. Therefore, the present study aimed to investigate the causal relationship between IS and FS using a two-sample Mendelian randomization method.

**Methods:**

Our research was divided into two stages: discovery and replication. The data were extracted from publicly available genome-wide association studies (GWAS). We selected a large sample of IS (*n* = 440, 328) and its subtypes (large-artery atherosclerotic stroke (LAS), cardioembolic stroke (CES), and stroke caused by small-vessel disease (SVS) and lacunar stroke (*n* = 254, 959) as exposure data. Additionally, we selected a large sample of FS as outcome data (*n* = 451, 099). Inverse variance weighting (IVW) was applied as the primary analysis method. The weighted median, MR-Egger, simple model, and weighted model were used as complementary analysis methods to assess causal effects. Moreover, heterogeneity was analyzed using Cochran's Q-test with IVW and MR-Egger. The MR-Egger intercept and MR-PRESSO analysis methods were used for pleiotropy testing. The stability of the results was also assessed using a leave-one-out analysis.

**Results:**

In the discovery stage, the IVW approach revealed an odds ratio (OR) of 1.207 with a 95% confidence interval (CI) of 1.027–1.417 and a *P*-value of 0.022. This suggests a causal association between IS levels and an increased risk of FS. In the subtype studies of IS, the findings were negative. However, during the replication stage, a significant causal link was found between selected lacunar strokes and FS with an OR of 1.252, a 95% CI of 1.105–1.419, and a *P*-value of 0.0004. All studies had no pleiotropy or heterogeneity, and the findings were robust.

**Conclusions:**

Our study confirmed the causal relationship between any IS level and increased risk of FS. Furthermore, the same results were obtained in the replication stage with lacunar stroke as an exposure factor. However, there was no direct causal relationship between the subtypes of IS and FS. Our study provides theoretical support for shoulder care for patients with IS.

## 1. Introduction

Frozen shoulder (FS), which is also referred to as adhesive capsulitis, has a prevalence rate of 2–5% in the population (1), more commonly in women aged 40–60 years (2). FS is characterized by a gradual reduction in shoulder motor function accompanied by shoulder pain (3). Currently, there is insufficient evidence to determine the most effective treatment for patients with FS, which continues to be a controversy (4). Studies have shown that 50% of patients with FS will still develop shoulder pain or stiffness for an average of 7 years after the onset of the disease (5). Additionally, only 39% of patients were observed to have a complete recovery at the 5 to 10-year follow-up (6). One survey found that more than 140,000 people over the age of 65 in the United States have FS, and it increases the burden of health care coverage for the elderly population in the United States (7). A recent study employing genome-wide data reveals that type I diabetes is a genetic risk factor for a frozen shoulder (8). However, the cause of periarthritis is still unclear; therefore, it is essential to actively explore the exact etiology of FS.

Stroke is a major contributor to global mortality rates and a significant factor in causing severe and long-lasting disabilities (9). Each year, there are over 795,000 cases of stroke in the United States, with ischemic strokes making up 87% of all instances. It is believed that the yearly expense associated with stroke in the United States is around 100 million dollars (10). The mortality rate of ischemic stroke (IS) has been decreasing in the last 2 years, but the rate of IS survivors and overall disability has been increasing yearly (11). Recently, several studies have shown a strong relationship between IS and FS. One study looking at patients with adhesive capsulitis at follow-up found that the risk ratio for stroke in patients with adhesive capsulitis was 1.46 times higher than in healthy controls (95% CI, 1.32–1.62; *P* < 0.001) (12). On the other hand, hemiplegic shoulder pain impacts ~22–23% of stroke survivors, and the number of individuals suffering from shoulder ailments such as frozen shoulder, scapular adhesions, and shoulder subluxation is on the rise annually (13, 14). Early identification of patients at high risk of developing or recurring IS and early interventions related to IS patients are essential. Therefore, we conducted a Mendelian randomization (MR) study to focus on the causal association between IS and FS.

From observational studies, it is suggested that IS and FS show a positive association (13, 15). However, the association between IS and FS risk may have been overestimated due to shortcomings such as reverse causality, small sample size, and confounding factors. In addition, whether IS subtypes have a differential effect on FS remains to be determined. Mendelian randomization (MR) analysis is a methodology akin to randomized controlled trials (16), in which single nucleotide polymorphisms (SNPs) are utilized as instrumental variables (IVs) to deduce causal links between exposure and outcome (17). MR analysis can minimize bias due to confounding variables and prevent interference from reverse causation since alleles segregate randomly during meiosis, independent of external factors, and genetic variations occur before disease onset (18, 19). This method has also been used more often in various clinical causality inferences. For example, Harry D. Green et al. demonstrated a causal relationship between type I diabetes and frozen shoulders by MR analysis. They concluded that obesity was not causally related to FS (8). Also, Ellervik et al. demonstrated a causal relationship between thyroid function and atrial fibrillation causal relationship (20). However, studies focusing on the association of IS with FS by MR analysis are not available for now.

Our study used a large-scale genome-wide association study (GWAS) database to assess the causal relationship between IS and its subtypes and FS through a two-sample MR study analysis.

## 2. Materials and methods

### 2.1. Study design

**Figure 1:**
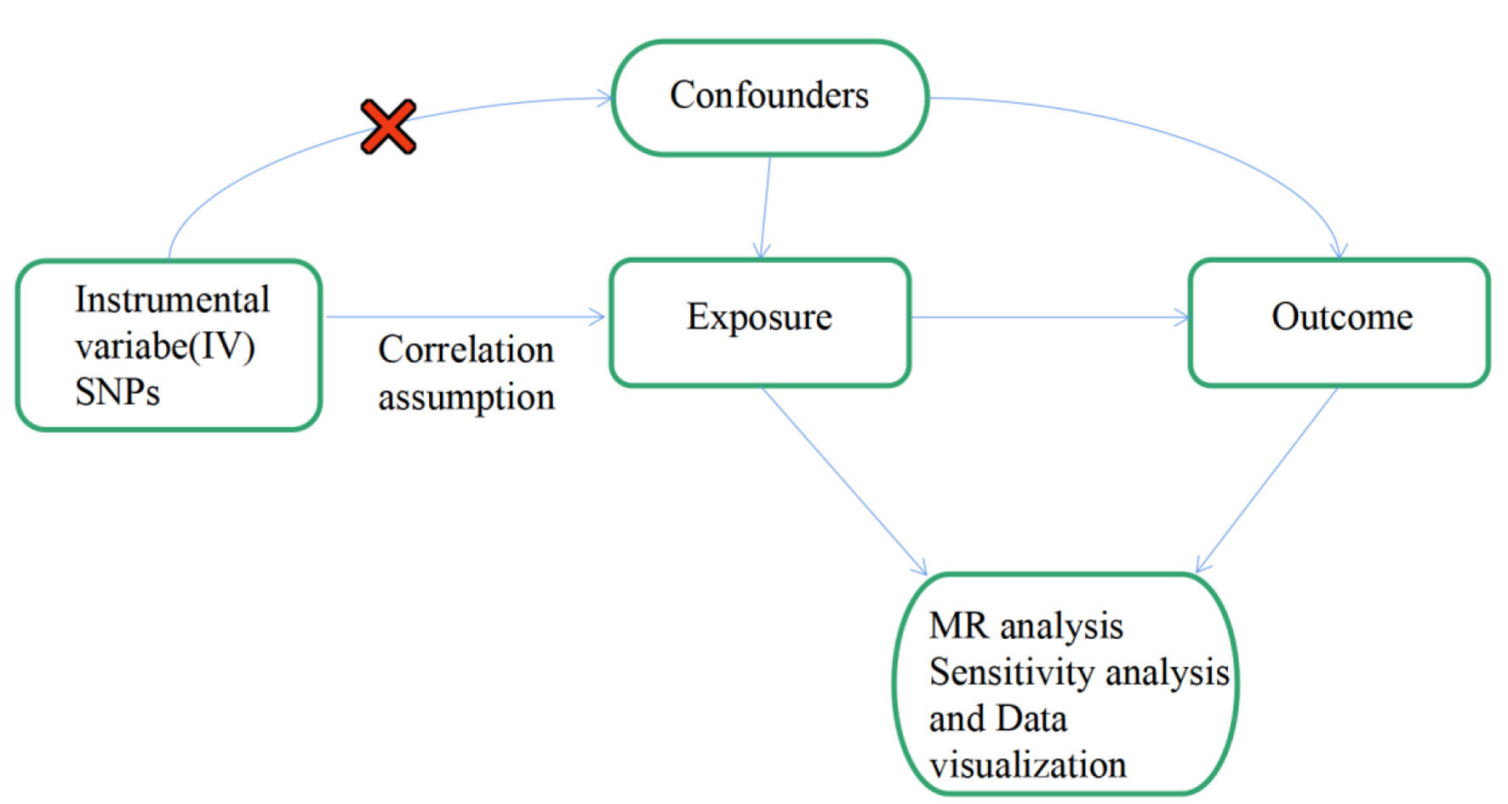
Flow chart: this is a schematic representation of the two-sample MR analysis of IS and its subtypes with FS. Three main hypotheses of the MR analysis. Hypothesis 1: genetic variation as an instrumental variable must have a valid association with exposure (IS and its subtypes and replication stage of lacunar stroke). Hypothesis 2: exposure-outcome confounders do not affect genetic variance. Hypothesis 3: genetic variation affects outcome (FS) only through exposure (IS and its subtypes and lacunar stroke at the replication stage), independent of other pathways. MR, Mendelian randomization; SNPs, single nucleotide polymorphisms; OR, odds ratio.

To be an effective tool for causal reasoning in MR studies, genetic variation must meet three core assumptions. Assumption 1: Genetic variation as an instrumental variable must be truly correlated with exposure (IS). Assumption 2: Genetic variation was not associated with exposure-outcome confounders. Assumption 3: Genetic variation affects outcome (FS) only through exposure (IS), independent of other pathways (18, 21). Figure 1 shows an overview of our study design. In this MR investigation, previously published and publicly available large-scale GWAS summary datasets were utilized. In the relevant original GWASs, all subjects supplied signed informed permission.

### 2.2. Genetic instrument selection (discovery)

IS is classified into three etiological subtypes: large-artery atherosclerotic stroke (LAS), cardioembolic stroke (CES), and stroke caused by small-vessel disease (SVS). The data on IS and its subtypes used in our study were obtained from a GWAS summary encompassing 520,000 individuals and carried out by multiple consortia, including the International Stroke Federation (22). The IS and subtypes were all European populations and involved more than 8 million single nucleotide polymorphisms (SNPs). In the discovery stage, we set genome-wide significance thresholds for IS (*P* < 5.00E-09) and its subtypes (*P* < 5.00E-06). We eliminated linkage disequilibrium (kb > 10,000 and r2 < 0.001) and excluded palindromic SNPs with moderate allele frequencies. In addition, we quantified the strength of the genetic instrument for all SNPs with an F-statistic calculated as (β2/se2) and as a follow-up analysis for instrumental variables (IVs) with an F-statistic higher than 10 (23). We used MR-PRESSO to screen the final IVs for the presence of abnormal SNPs. Finally, the instrumental variables employed for IS, LAS, CES, and SVS IVs encompassed 9, 28, 28, and 31 SNPs, respectively (Supplementary Table 1).

#### 2.2.1. Genetic instrument selection (replication)

In the replication stage, we relied on a GWAS meta-analysis of a European population that included 6,030 cases and 248,929 controls, which covered almost 7 million SNPs. The meta-analysis combined data from prior GWAS, the International Stroke Genetics Consortium, and the UK DNA Luminal Stroke Study, providing an abundant and comprehensive dataset (24). We set genome-wide significance thresholds (*P* < 5.00E-08), eliminated linkage disequilibrium (kb > 10,000 and r2 < 0.001), and excluded palindromic SNPs with medium allele frequencies. Finally, three SNPs were left as instrumental variables for the validation phase of the analysis. Detailed information on the IVs is provided in Supplementary Table 1.

### 2.3. FS GWAS selection

The data on FS were collected through a combined GWAS analysis of FinnGen and the UK Biobank, incorporating data from 10,104 cases identified through inpatient, surgical, and primary care codes (8). This data is the most comprehensive GWAS on FS, comprising individuals of European ancestry, and involving 451,099 participants and over 15 million SNPs (8).

### 2.4. MR method selection

We performed MR analysis using genetic data extracted from GWAS summary data. The TwoSampleMR package (version 0.5.6) in R (version 4.1.2) was used to analyze the data between exposures and outcomes (25). The primary method of analysis used was the inverse variance weighted (IVW) with random effects. In addition, several supplementary analysis methods were used, including MR-Egger, weighted median, simple mode, and weighted mode. The threshold of statistical significance was P < 0.05. The IVW method assumes that all SNPs included in the analysis can be used as valid IVs, when this method can provide greater help for the analysis (26). The weighted median gives accurate estimates based on the assumption that the number of valid IVs is 50% (27). The MR-Egger regression assumes that all IVs are invalid IVs, and the estimation accuracy of this method is relatively low (28, 29). The simple mode and weighted mode are not as robust as IVW, but they provide a means to test the consistency and stability of the results (30).

### 2.5. Sensitivity analysis

In addition, we will perform a series of sensitivity analyses, including heterogeneity and pleiotropy. IVW and MR-Egger regression were used to test for heterogeneity, and Q statistics were produced to quantify it (31). In cases where heterogeneity was present, we utilized IVW with random effects to conduct the analysis. Horizontal pleiotropy is essential for our study because being affected by horizontal pleiotropy may lead to unstable effect estimates. The MR-Egger intercept method calculates the intercept term from linear regression analysis to estimate the likelihood of horizontal pleiotropy (32). The MR-PRESSO examination assesses the overall pleiotropy of the study and screens for abnormal SNPs that may have horizontal pleiotropy (33). We increased the number of distributions to 5,000 in the MR-PRESSO analysis using software and then conducted a global test to detect pleiotropy in the study. The robustness of the MR analysis results was further evaluated by comparing the impacts before and after the removal of aberrant SNPs (34).

### 2.6. Data visualization

To prevent bias in the results caused by individual SNP pleiotropy, we conducted a leave-one-out analysis to examine the impact of each SNP on the causal relationship between IS and FS risk (35). The presence or absence of directional pleiotropy was assessed by funnel plot and MR-Egger intercept (36). The forest plots were used to visualize the effect estimates between genetic variants and IS, and the MR-Egger regression with IVW was utilized to calculate the combined effects (33).

## 3. Results

The causal relationship between the levels of IS and its subtypes and FS risk was explored by two-sample MR analysis and validated using lacunar stroke data. Our MR results revealed a causal association between genetic susceptibility to IS and increased risk of FS, but no association was found in subtypes of IS. Moreover, a causal association between lacunar stroke and increased risk of FS was also revealed in the replication phase.

### 3.1. MR analysis in the discovery stage

**Figure 2:**
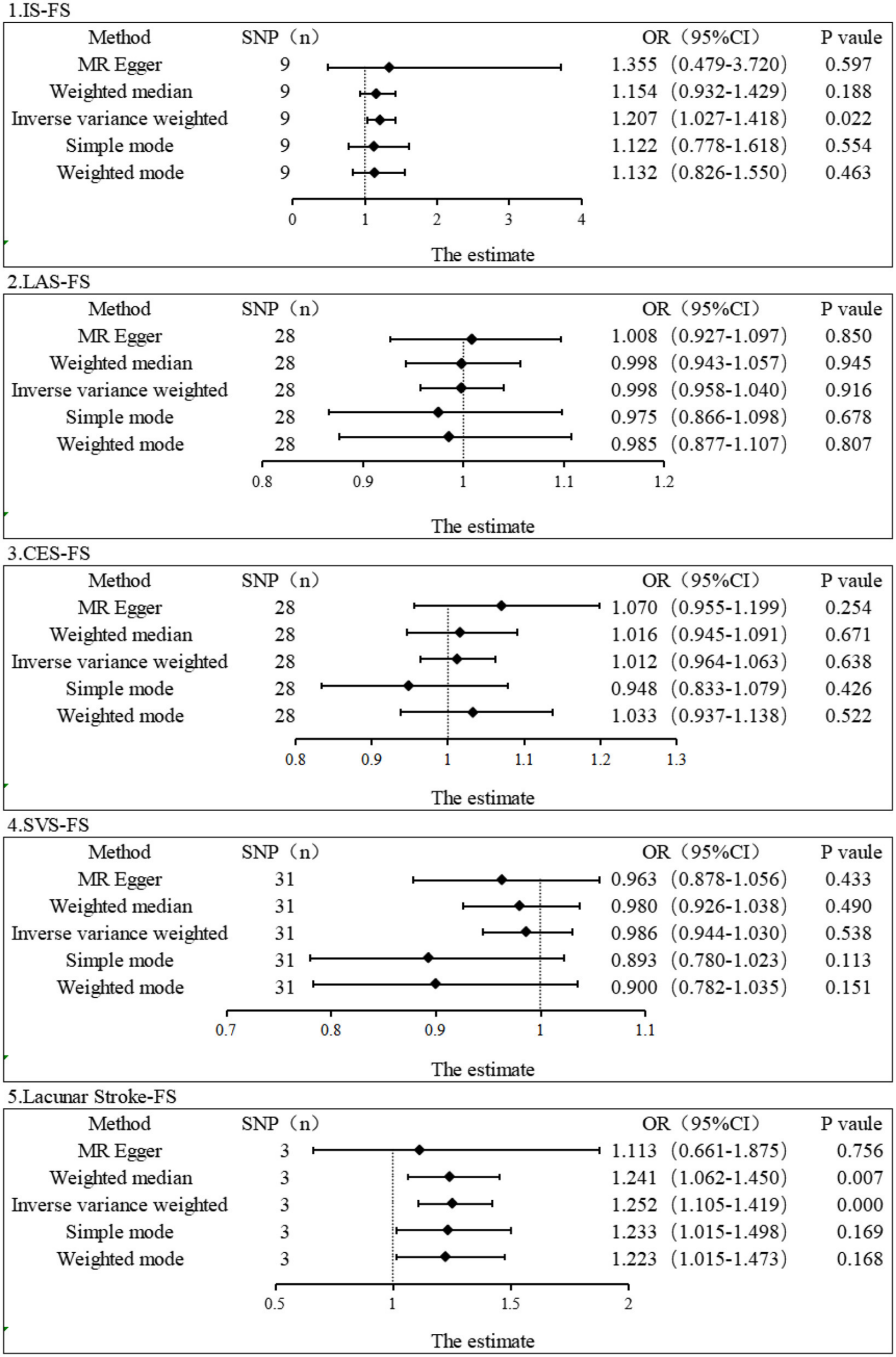
Estimation of the causal relationship between IS and its subtypes and FS using different MR methods. An OR value > 1 suggests that the exposure indicator is a risk factor, while the opposite is a protective factor.

**Figure 3:**
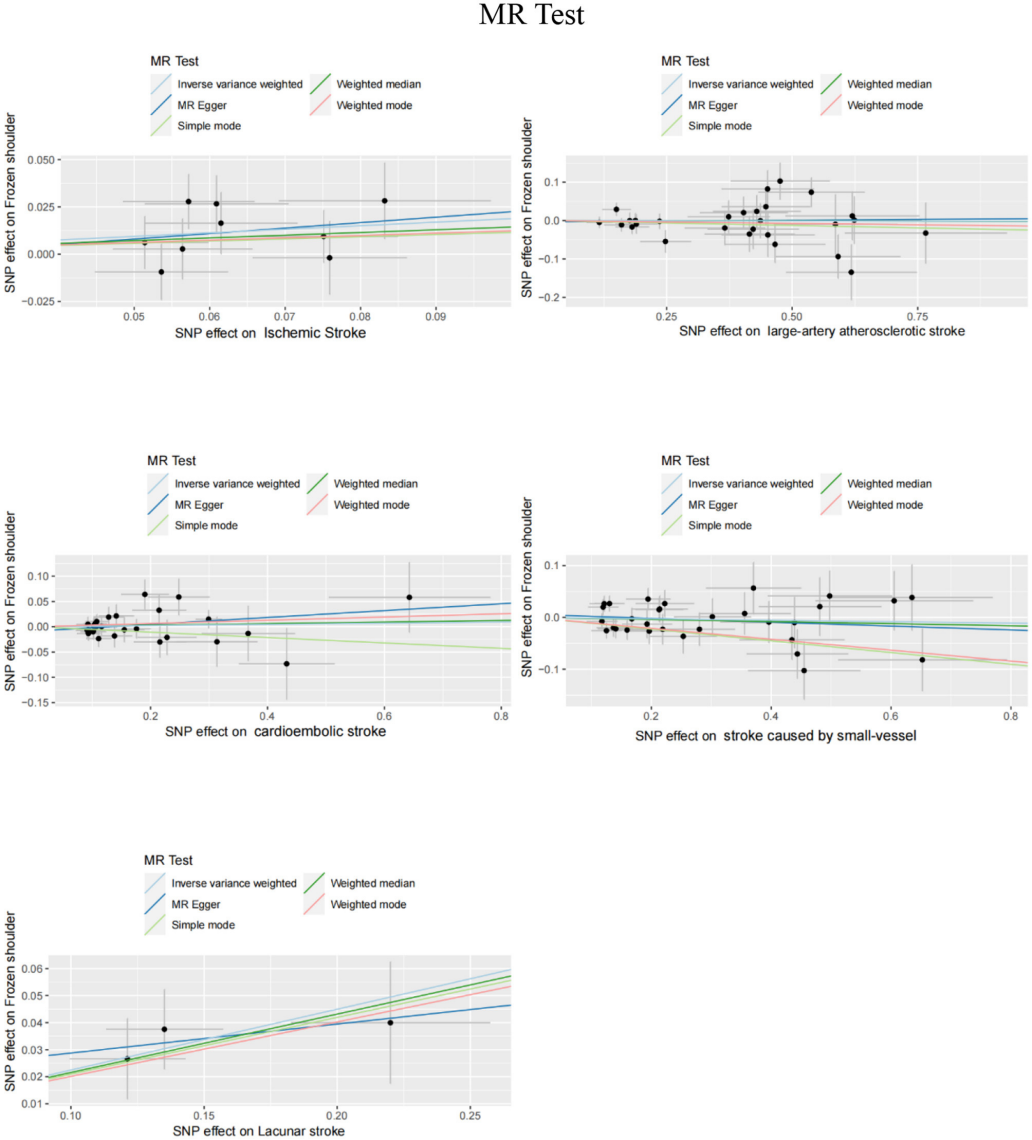
Scatter plot of genetic correlation between IS and its subtypes and FS by different MR analysis methods.

We carefully examined our IVs and then used MR analysis to evaluate the summarized data for IS and its subtypes. The primary MR analysis method showed a significant correlation between IS and FS risk (IVW, OR = 1.207, 95% CI, 1.027–1.417, *P* = 0.022). However, no correlation was found between subtypes of IS and FS risk (IVW: LAS, OR = 0.998, 95% CI, 0.958–1.040, *P* = 0.916; CES, OR = 1.012, 95% CI, 0.964–1.063, *P* = 0.638; SVS, OR = 0.986, 95% CI, 0.944–1.030, *P* = 0.538) (Figure 2). In the IS and FS risk study, the ORs obtained by converting the relative risk ratios were all > 1, suggesting that IS may be a risk factor for FS. MR-Egger regression and IVW analysis were used to detect heterogeneity. MR-Egger regression (Cochran's Q = 5.768, *P* = 0.567) and IVW (Cochran's Q = 5.806, *P* = 0.669) indicated that there was no heterogeneity in the study (Table 1). Additionally, both the MR-Egger intercept examination (intercept = −0.006 and *P*-value = 0.850) and the MRPRESSO global test (*P*-value = 0.766) showed no signs of pleiotropy (Table 2). Figure 3 shows scatter plots of the causal estimates between IS and its subtypes and FS using IVW, MR-Egger, weighted median, weighted mode, and simple mode analyses. We used the leave-one-out method to remove SNPs one by one to determine whether the causal association was caused by a single IV, which ultimately showed that the analysis of IS with FS was more robust (Supplementary Figure 1). Supplementary Figures 2, 3 show the funnel plot and forest plot, respectively, between the analysis of IS and its subtypes with FS.

**Table 1:** Heterogeneity test of MR studies.

**Sensitivity analysis**							
**Heterogeneity test**							
**Exposure**	**Outcome**	**Heterogeneity test (MR-Egger)**			**Heterogeneity test (IVW)**		
		**Cochran's Q**	**Q_df**	***P***	**Cochran's Q**	**Q_df**	***P***
IS	FS	5.768	7	0.567	5.806	8	0.669
LAS	FS	30.394	26	0.252	30.486	27	0.293
CES	FS	19.402	26	0.819	20.544	27	0.807
SVS	FS	35.431	29	0.191	35.834	30	0.214
Lacunar stroke	FS	0.208	1	0.648	0.416	2	0.812

**Table 2:** Pleiotropy test of MR studies.

**Sensitivity analysis**				
**Heterogeneity test**				
**Exposure**	**Outcome**	**Horizontal pleiotropy test (MR-Egger)**		**MR-PRESSO**
		**Intercept**	***P***	**Global test** ***P*****-value**
IS	FS	−0.006	0.850	0.766
LAS	FS	−0.003	0.782	0.287
CES	FS	−0.009	0.295	0.980
SVS	FS	0.006	0.570	0.319
Lacunar stroke	FS	0.018	0.728	–

### 3.2. MR analysis in the replication stage

As lacunar stroke represents a significant indication of cerebral small vessel disease and comprises 25% of IS cases, we opted for lacunar stroke as our replicated exposure data stage (37). We finally obtained 3 SNPs as IVs for replication stage MR analysis by setting significant genome-wide thresholds and eliminating linkage disequilibrium, among other manipulations. Using IVW and weighted median, we found that lacunar stroke level was associated with increased risk of FS (IVW, OR = 1.252, 95% CI: 1.105–1.419, *P* = 0.0004; Weighted median, OR = 1.241, 95% CI: 1.062–1.450, *P* = 0.007) (Figure 2). No significant results were reported in other MR methods, including MR-Egger (OR = 1.355, 95% CI: 0.479–3.720, *P* = 0.597), simple mode (OR = 1.122, 95% CI: 0.778–1.618, *P* = 0.554), and weighted mode (OR = 1.132, 95% CI: 0.826–1.550, *P* = 0.463). Nevertheless, the conversion of odds ratio values indicated a consistent direction, indicating that the level of lacunar stroke is a risk factor for FS. Additionally, the MR-Egger intercept test showed no indication of pleiotropy (Intercept = 0.018, and *P* = 0.728). The number of final IVs was too small and did not allow for the MRPRESSO test. Our examination of the PhenoScanner database (www.phenoScanner.medschl.cam.ac.uk) failed to reveal any SNPs associated with potential confounders for FS, confirming the robustness of our findings. Supplementary Figure 1 shows scatter plots of our single causal estimates for the 3 SNPs using the five MR analysis methods. Supplementary Figures 2, 3 show the funnel plot and forest plot, respectively, between lacunar stroke and FS analysis.

## 4. Discussion

Damage to the blood vessels in the brain often causes abnormalities in the central nervous system, which can result in somatosensory abnormalities, manifesting symptoms such as shoulder and low back pain. In addition, stroke often causes hemiplegia of one limb, resulting in hemiplegic shoulder pain. According to a study that followed 416 patients for a year, roughly 33% of the patients experienced shoulder pain following a stroke, and the majority of these patients reported moderate to severe levels of pain (38). In a recent study, the frequency of shoulder pain after stroke was about 30%, and the onset of hemiplegic shoulder pain was of long duration (39). According to a multicenter study, shoulder pain symptoms are common in stroke patients, and FS is the primary cause of shoulder pain following hemiplegia (40).

However, the causal relationship between IS and FS remains unclear. Most of the previous epidemiological studies were case-control designs or cross-sectional studies with a hazy chronological order that failed to clarify causality. Furthermore, past observational studies were plagued by insufficient sample numbers, difficulty avoiding reverse causation, and confounding factors. Therefore, we performed an MR analysis to explore the relationship between IS and FS from a genetic perspective.

In the current investigation, we discovered a causative relationship between IS and FS and confirmed this association by observing the same causal relationship in patients with lacunar stroke. However, no connection was discovered in the three most frequent IS subtypes. We believe that more research is needed to analyze our findings, maybe due to the small sample size of IS subtypes. The analysis of a larger sample of IS subtypes may be pursued in the future. The risk of developing FS is elevated with any IS, likely due to the somatosensory abnormalities resulting from lesions in the central nervous system post-stroke (41). On the other hand, stroke frequently causes paralysis in one limb, which increases the likelihood of joint effusions and muscle adhesions over time, ultimately leading to the development of FS (14, 42). Shoulder pain is a typical consequence of post-stroke hemiplegia patients. Shoulder pain following a stroke can prolong rehabilitation and hospitalization of the affected limb, hindering daily activities and impeding the recovery of upper limb and hand function. This reminds us to pay attention to shoulder care for stroke patients and to pay timely attention to whether the patient has a limited range of motion in the shoulder. In the daily care of IS patients, the patient's shoulder should be moved regularly. Moreover, the shoulder should also be the focus of our treatment when using physical therapy treatments such as acupuncture and massage.

The advantages of our two-sample MR study are as follows. First, we used the MR analysis method, using SNPs with high association strength (*F* > 10) as instrumental variables, and the experimental design was similar to randomized controlled trials. Randomized controlled trials are commonly employed in clinical practice and are considered to provide a high level of evidence. However, they have drawbacks, such as being costly and having limited sample sizes. The MR study approach effectively avoids reversing causality and confounding factors. Second, the data we used were all from the GWAS database, which is European population samples, effectively reducing the bias of population heterogeneity. Third, we validated the results of the discovery phase to confirm that the results obtained were reliable. The results of our analysis may have implications for healthcare policy. Revealing a causal relationship between IS and FS may influence public health policies regarding prevention and treatment.

Nonetheless, our study does have certain limitations. First, the leave-one-out method for the subtypes of IS in this study needed to be revised, possibly due to the small sample size. Our study found a causal association between lacunar stroke on the increased risk of FS during the replication phase but did not find an association in subtypes of IS. This implies that we should interpret the correlation between FS and IS subtypes with caution and investigate their association in future studies with larger sample sizes. Second, the prevalence of IS and FS differed between males and females. However, our data were all obtained from public databases, which did not allow for factor-specific subgroup analyses such as age and sex. Third, all subjects included in the GWAS data were of European descent, and further research is necessary to determine whether the findings can be extrapolated to other populations. Fourth, increasing the sample size of GWAS can improve the strength of IV, and we need larger scale GWAS for in-depth study.

## 5. Conclusion

In conclusion, our MR study supports a causal association between IS and increased FS risk, but no relationship was found in IS subtypes.

## Data availability statement

The original contributions presented in the study are included in the article/Supplementary material, further inquiries can be directed to the corresponding authors.

## Author contributions

XL, ZH, FL, SL, HG, JD, and XD: study design, data collection, and statistical analysis. J-HQ, QN, and JL: supervision. XL, ZH, and FL: writing—original draft. SL, HG, J-HQ, QN, and JL: writing—review and editing. All authors contributed to the article and approved the submitted version.
