# Supplementary material for: Causal relationship between ischemic stroke and its subtypes and frozen shoulder: a two-sample Mendelian randomization analysis
Source: Front Neurol. 2023 May 18;14:1178051. doi: 10.3389/fneur.2023.1178051 (PMC10233007; doi:10.3389/fneur.2023.1178051)
Supplement: Supplementary file 1 [file Data_Sheet_1.ZIP › Supplementary Materials Figure 3.docx]

Supplementary Figure 3：


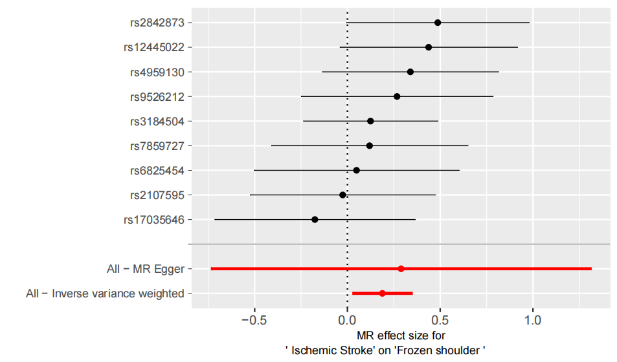

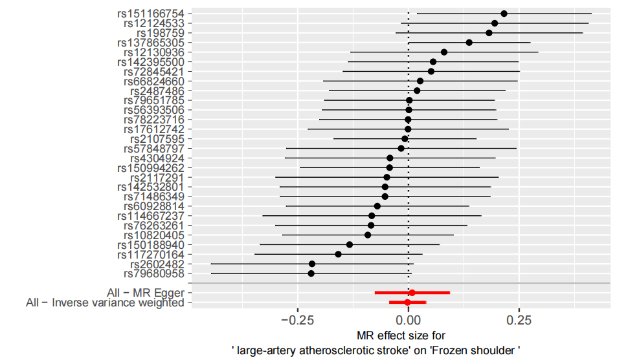

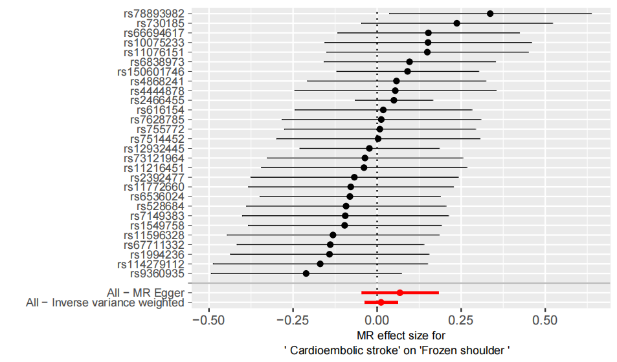

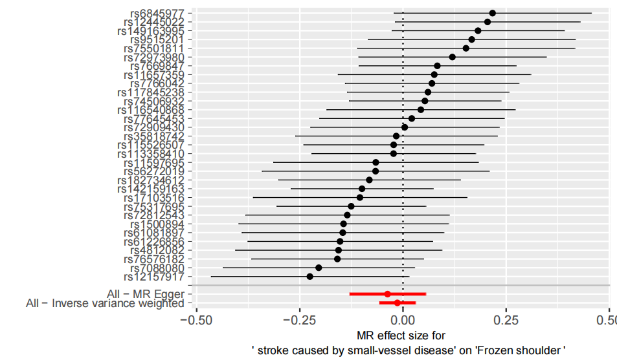

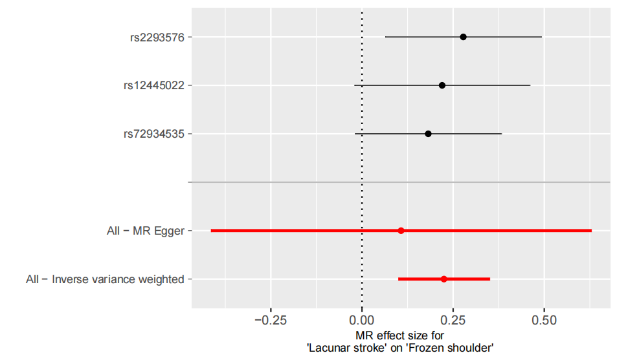


Supplementary Figure 3: Forest plots of the causal effects of IS and its subtypes with FS and associated SNPs. Red and black dots/lines indicate causal estimates of risk between IS and its subtypes and FS.
