# Supplementary material for: Causal relationship between ischemic stroke and its subtypes and frozen shoulder: a two-sample Mendelian randomization analysis
Source: Front Neurol. 2023 May 18;14:1178051. doi: 10.3389/fneur.2023.1178051 (PMC10233007; doi:10.3389/fneur.2023.1178051)
Supplement: Supplementary file 1 [file Data_Sheet_1.ZIP › Supplementary Materials Table 1.docx]

Supplementary Materials Table 1:

Instrumental Variable

| IS-FS | | | | | | | | |
| --- | --- | --- | --- | --- | --- | --- | --- | --- |
| SNP | Chr | EA | OA | Beta | Eaf | SE | P | F |
| rs12445022 | 16 | A | G | 0.0609 | 0.3057 | 0.0095 | 1.28E-10 | 41.09 |
| rs17035646 | 1 | A | G | 0.0536 | 0.405 | 0.0088 | 1.34E-09 | 37.10 |
| rs2107595 | 7 | A | G | 0.0759 | 0.2264 | 0.0102 | 9.25E-14 | 55.37 |
| rs2842873 | 1 | T | C | -0.057 | 0.3955 | 0.0087 | 4.86E-11 | 43.23 |
| rs3184504 | 12 | C | T | -0.075 | 0.5479 | 0.0098 | 2.17E-14 | 58.73 |
| rs4959130 | 6 | A | G | 0.0832 | 0.1372 | 0.014 | 2.83E-09 | 35.32 |
| rs6825454 | 4 | C | T | 0.0564 | 0.3078 | 0.0092 | 7.43E-10 | 37.58 |
| rs7859727 | 9 | T | C | 0.0514 | 0.5355 | 0.0084 | 1.05E-09 | 37.44 |
| rs9526212 | 13 | G | A | 0.0615 | 0.7614 | 0.0101 | 9.19E-10 | 37.08 |
|  |  |  |  |  |  |  |  |  |
| LAS-FS | | | | | | | | |
| SNP | Chr | EA | OA | Beta | Eaf | SE | P | F |
| rs10820405 | 9 | A | G | -0.181 | 0.1847 | 0.0331 | 4.51E-08 | 29.97 |
| rs114667237 | 1 | A | G | 0.4516 | 0.0195 | 0.0925 | 1.05E-06 | 23.84 |
| rs117270164 | 19 | G | A | 0.5915 | 0.0152 | 0.1234 | 1.65E-06 | 22.98 |
| rs12124533 | 1 | T | C | 0.1499 | 0.2499 | 0.0278 | 6.73E-08 | 29.07 |
| rs12130936 | 1 | C | T | 0.4477 | 0.0272 | 0.0882 | 3.82E-07 | 25.77 |
| rs137865305 | 3 | A | G | 0.5381 | 0.0346 | 0.1056 | 3.47E-07 | 25.97 |
| rs142395500 | 18 | A | G | 0.4295 | 0.0241 | 0.0869 | 7.61E-07 | 24.43 |
| rs142532801 | 10 | T | C | 0.3658 | 0.0273 | 0.0798 | 4.55E-06 | 21.01 |
| rs150188940 | 3 | G | A | 0.4659 | 0.0195 | 0.0994 | 2.73E-06 | 21.97 |
| rs150994262 | 1 | A | G | 0.766 | 0.012 | 0.1609 | 1.94E-06 | 22.66 |
| rs151166754 | 18 | T | C | 0.4759 | 0.0235 | 0.0985 | 1.35E-06 | 23.34 |
| rs17612742 | 4 | C | T | 0.1764 | 0.1378 | 0.0361 | 1.05E-06 | 23.88 |
| rs198759 | 11 | G | A | 0.451 | 0.0228 | 0.0918 | 8.97E-07 | 24.14 |
| rs2107595 | 7 | A | G | 0.2358 | 0.1677 | 0.0319 | 1.44E-13 | 54.64 |
| rs2117291 | 2 | C | T | -0.19 | 0.907 | 0.0415 | 4.95E-06 | 20.85 |
| rs2487486 | 10 | T | G | -0.62 | 0.9885 | 0.133 | 3.16E-06 | 21.72 |
| rs2602482 | 11 | A | G | 0.6177 | 0.0128 | 0.1301 | 2.04E-06 | 22.54 |
| rs4304924 | 13 | A | G | 0.1158 | 0.5689 | 0.0251 | 3.95E-06 | 21.28 |
| rs56393506 | 6 | T | C | 0.1878 | 0.1639 | 0.0355 | 1.26E-07 | 27.99 |
| rs57848797 | 9 | C | T | 0.5861 | 0.0151 | 0.1227 | 1.79E-06 | 22.82 |
| rs60928814 | 11 | G | A | -0.16 | 0.2188 | 0.0345 | 3.44E-06 | 21.53 |
| rs66824660 | 14 | A | C | 0.3734 | 0.0298 | 0.0814 | 4.49E-06 | 21.04 |
| rs71486349 | 11 | T | G | 0.4222 | 0.0208 | 0.0905 | 3.07E-06 | 21.76 |
| rs72845421 | 2 | T | C | 0.4032 | 0.026 | 0.0873 | 3.83E-06 | 21.33 |
| rs76263261 | 18 | C | T | 0.4149 | 0.0258 | 0.0894 | 3.43E-06 | 21.54 |
| rs78223716 | 13 | G | A | 0.4367 | 0.023 | 0.0937 | 3.17E-06 | 21.72 |
| rs79651785 | 12 | A | G | 0.6238 | 0.0148 | 0.1336 | 3.00E-06 | 21.80 |
| rs79680958 | 4 | A | G | 0.2475 | 0.0754 | 0.0503 | 8.60E-07 | 24.21 |
|  |  |  |  |  |  |  |  |  |
| CES-FS | | | | | | | | |
| SNP | Chr | EA | OA | Beta | Eaf | SE | P | F |
| rs10075233 | 5 | A | G | 0.1412 | 0.1207 | 0.0296 | 1.89E-06 | 22.76 |
| rs11076151 | 16 | A | C | 0.1282 | 0.1617 | 0.0259 | 7.39E-07 | 24.50 |
| rs11216451 | 11 | G | A | 0.155 | 0.0847 | 0.0339 | 4.81E-06 | 20.91 |
| rs114279112 | 2 | G | T | 0.4327 | 0.0191 | 0.0816 | 1.14E-07 | 28.12 |
| rs11596328 | 10 | T | G | 0.1382 | 0.1193 | 0.0296 | 3.04E-06 | 21.80 |
| rs11772660 | 7 | G | A | 0.0916 | 0.3928 | 0.0198 | 3.57E-06 | 21.40 |
| rs12932445 | 16 | C | T | 0.1758 | 0.1805 | 0.0245 | 6.88E-13 | 51.49 |
| rs150601746 | 1 | T | G | 0.6427 | 0.0112 | 0.1379 | 3.15E-06 | 21.72 |
| rs1549758 | 7 | C | T | -0.102 | 0.6868 | 0.0216 | 2.61E-06 | 22.12 |
| rs1994236 | 5 | G | A | 0.0935 | 0.4474 | 0.0198 | 2.23E-06 | 22.30 |
| rs2392477 | 7 | C | T | -0.094 | 0.3535 | 0.0201 | 3.17E-06 | 21.64 |
| rs2466455 | 4 | T | C | -0.299 | 0.7826 | 0.0222 | 2.75E-41 | ##### |
| rs4444878 | 4 | A | C | 0.0928 | 0.4 | 0.0196 | 2.27E-06 | 22.42 |
| rs4868241 | 5 | T | C | -0.105 | 0.5895 | 0.0197 | 1.05E-07 | 28.30 |
| rs528684 | 11 | A | C | -0.228 | 0.9571 | 0.0499 | 4.87E-06 | 20.88 |
| rs616154 | 9 | T | C | 0.1035 | 0.5112 | 0.0193 | 7.98E-08 | 28.76 |
| rs6536024 | 4 | C | T | 0.1023 | 0.53 | 0.0195 | 1.46E-07 | 27.52 |
| rs66694617 | 4 | T | C | 0.2145 | 0.0546 | 0.0457 | 2.65E-06 | 22.03 |
| rs67711332 | 10 | A | G | 0.2159 | 0.051 | 0.0448 | 1.46E-06 | 23.22 |
| rs6838973 | 4 | T | C | -0.108 | 0.4341 | 0.0196 | 3.58E-08 | 30.31 |
| rs7149383 | 14 | A | G | 0.3135 | 0.0226 | 0.0687 | 4.98E-06 | 20.82 |
| rs730185 | 5 | C | T | 0.2482 | 0.0389 | 0.0523 | 2.04E-06 | 22.52 |
| rs73121964 | 12 | G | A | 0.3668 | 0.0224 | 0.0794 | 3.85E-06 | 21.34 |
| rs7514452 | 1 | T | C | -0.116 | 0.8051 | 0.0236 | 8.78E-07 | 24.20 |
| rs755772 | 5 | C | T | -0.108 | 0.2705 | 0.0224 | 1.58E-06 | 23.07 |
| rs7628785 | 3 | A | G | -0.102 | 0.2731 | 0.022 | 3.34E-06 | 21.62 |
| rs78893982 | 9 | G | T | 0.1902 | 0.0711 | 0.0405 | 2.63E-06 | 22.06 |
| rs9360935 | 6 | C | A | 0.1111 | 0.265 | 0.0215 | 2.31E-07 | 26.70 |
|  |  |  |  |  |  |  |  |  |
| SVS-FS | | | | | | | | |
| SNP | Chr | EA | OA | Beta | Eaf | SE | P | F |
| rs113358410 | 5 | C | T | 0.3961 | 0.0262 | 0.0811 | 1.06E-06 | 23.85 |
| rs115526507 | 2 | C | T | 0.4388 | 0.0216 | 0.0886 | 7.32E-07 | 24.53 |
| rs11597695 | 10 | T | C | 0.1175 | 0.3247 | 0.0254 | 3.81E-06 | 21.40 |
| rs116540868 | 3 | A | G | 0.4811 | 0.0168 | 0.1018 | 2.31E-06 | 22.33 |
| rs11657359 | 17 | A | C | -0.214 | 0.0881 | 0.0465 | 4.14E-06 | 21.16 |
| rs117845238 | 16 | G | T | 0.6349 | 0.0118 | 0.1341 | 2.20E-06 | 22.42 |
| rs12157917 | 22 | T | C | 0.4549 | 0.0183 | 0.0934 | 1.12E-06 | 23.72 |
| rs12445022 | 16 | A | G | 0.1301 | 0.3367 | 0.0244 | 9.26E-08 | 28.43 |
| rs142159163 | 1 | T | C | 0.4345 | 0.0303 | 0.0873 | 6.52E-07 | 24.77 |
| rs149163995 | 2 | T | C | -0.195 | 0.1256 | 0.0368 | 1.22E-07 | 27.93 |
| rs1500894 | 8 | C | T | 0.2533 | 0.0448 | 0.0553 | 4.59E-06 | 20.98 |
| rs17103516 | 14 | A | G | 0.2189 | 0.0716 | 0.0469 | 3.09E-06 | 21.78 |
| rs182734612 | 19 | G | A | 0.2802 | 0.0519 | 0.0587 | 1.84E-06 | 22.79 |
| rs35818742 | 10 | C | T | 0.1674 | 0.1344 | 0.0329 | 3.65E-07 | 25.89 |
| rs4812082 | 20 | C | T | -0.141 | 0.8099 | 0.0298 | 2.30E-06 | 22.26 |
| rs56272019 | 8 | C | A | 0.1927 | 0.0849 | 0.0414 | 3.29E-06 | 21.67 |
| rs61081897 | 7 | T | C | 0.1368 | 0.2149 | 0.0281 | 1.08E-06 | 23.70 |
| rs61226856 | 16 | T | C | 0.1595 | 0.1971 | 0.0315 | 4.25E-07 | 25.64 |
| rs6845977 | 4 | C | T | 0.1213 | 0.3273 | 0.0242 | 5.40E-07 | 25.12 |
| rs7088080 | 10 | A | C | 0.1251 | 0.6595 | 0.0252 | 6.78E-07 | 24.64 |
| rs72812543 | 16 | T | C | 0.1965 | 0.094 | 0.0419 | 2.76E-06 | 21.99 |
| rs72909430 | 11 | A | C | 0.3018 | 0.042 | 0.0639 | 2.29E-06 | 22.31 |
| rs72973980 | 11 | T | C | 0.2224 | 0.0768 | 0.0484 | 4.31E-06 | 21.11 |
| rs74506932 | 20 | T | C | 0.6051 | 0.0134 | 0.1312 | 3.98E-06 | 21.27 |
| rs75317695 | 1 | C | A | 0.6522 | 0.0129 | 0.1404 | 3.40E-06 | 21.58 |
| rs75501811 | 1 | G | A | 0.3704 | 0.0214 | 0.079 | 2.78E-06 | 21.98 |
| rs76576182 | 7 | G | A | 0.4436 | 0.0212 | 0.0844 | 1.46E-07 | 27.62 |
| rs7669847 | 4 | C | T | 0.4975 | 0.0248 | 0.1035 | 1.54E-06 | 23.10 |
| rs77645453 | 4 | G | A | 0.3557 | 0.0303 | 0.0762 | 3.07E-06 | 21.79 |
| rs7766042 | 6 | C | T | 0.2129 | 0.1016 | 0.0397 | 7.97E-08 | 28.76 |
| rs9515201 | 13 | C | A | -0.12 | 0.6937 | 0.0247 | 1.34E-06 | 23.41 |
|  |  |  |  |  |  |  |  |  |
| lacunar stroke-FS | | | | | | | | |
| SNP | Chr | EA | OA | Beta | Eaf | SE | P | F |
| rs12445022 | 16 | A | G | 0.1212 | NA | 0.0217 | 2.48E-08 | 31.20 |
| rs2293576 | 11 | A | G | -0.135 | NA | 0.0219 | 7.22E-10 | 38.06 |
| rs72934535 | 2 | C | T | -0.22 | NA | 0.0373 | 3.72E-09 | 34.79 |

Supplementary Materials Table 1: SNP: single nucleotide polymorphism; EA: effect allele; OA: non-effect allele; Chr: chromosome; EAF: effect allele frequency; Beta was obtained by allele-related effects; SE: standard error. Beta, SE, and P are SNP summary statistics; F: F-statistic; IS: Ischemic Stroke; LAS: large-artery atherosclerotic stroke; CES: cardioembolic stroke; SVS: stroke caused by small-vessel disease.
