# Supplementary material for: Causal relationship between ischemic stroke and its subtypes and frozen shoulder: a two-sample Mendelian randomization analysis
Source: Front Neurol. 2023 May 18;14:1178051. doi: 10.3389/fneur.2023.1178051 (PMC10233007; doi:10.3389/fneur.2023.1178051)
Supplement: Supplementary file 1 [file Data_Sheet_1.ZIP › Supplementary Materials Figure 1.docx]

Supplementary Figure 1:

Leave-one-out sensitivity analysis


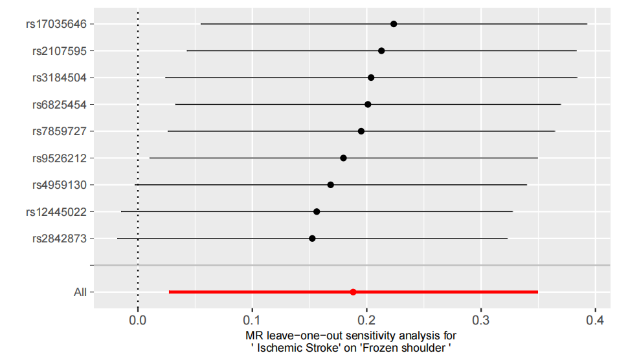

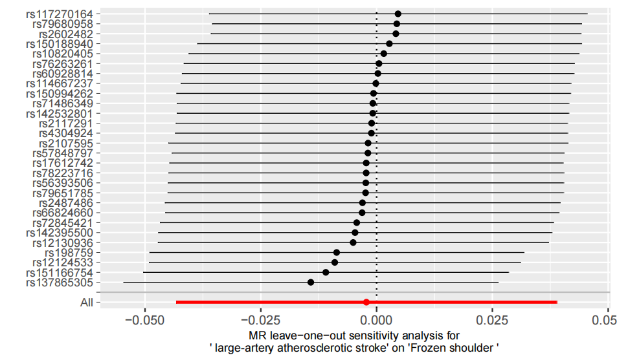

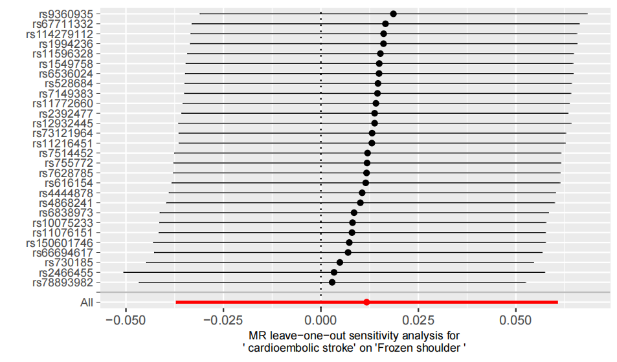

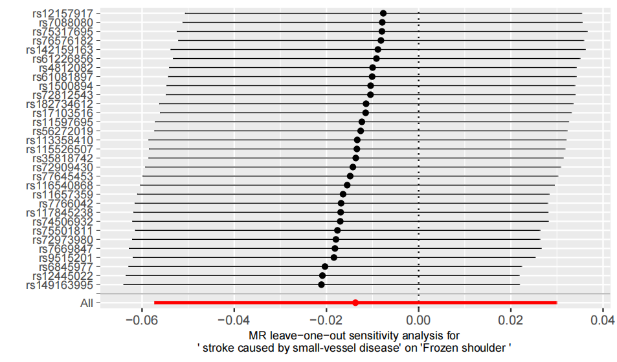

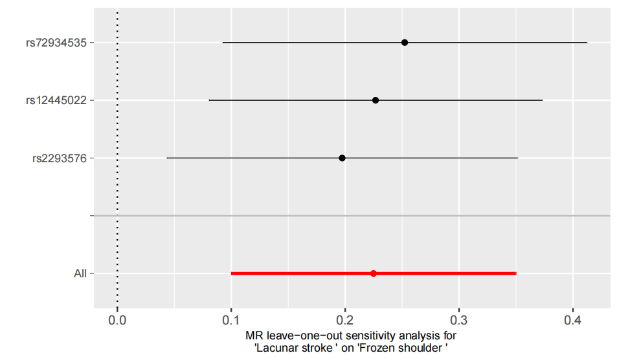


Supplementary Figure 1: Leave-one-out sensitivity analysis between IS and its Subtypes and FS. Red lines represent estimates from IVW tests. IVW: inverse variance weighted.
